# In-contest body acceleration profiles for the judo male and female weight divisions

**DOI:** 10.3389/fspor.2024.1372314

**Published:** 2024-03-11

**Authors:** Luis Santos, Peter A. Federolf, Friedemann Schneider, Elena Pocecco, Javier Fernández-Río, Eliseo Iglesias-Soler, Eduardo Carballeira-Fernández, Sugoi Uriarte, Xurxo Dopico-Calvo

**Affiliations:** ^1^ University of León, Department of Physical Education and Sport, León, Spain; ^2^ Performance and Health Group, Department of Physical Education and Sport, University of A Coruna, A Coruna, Spain; ^3^ Department of Sport Science, University of Innsbruck, Innsbruck, Austria; ^4^ Department of Orthopedics and Traumatology, Medical University of Innsbruck, Innsbruck, Austria; ^5^ Department of Educational Sciences, University of Oviedo, Oviedo, Spain; ^6^ Doctorate School, Universitat Politècnica de València, Valencia, Spain

**Keywords:** human motion assessment, physical activity’s patterns, combat sports, mechanical parameters, training

## Abstract

**Introduction:**

This study aimed to determine the body accelerations (BA) profile of the judo contest of the male and female weight divisions and to ascertain the involvement of the vertical, mediolateral and anteroposterior axes in it.

**Methods:**

Forty-eight male and forty-eight female national and international level athletes (some of them medalists in World, European and national championships) participated in a 5-min simulated contest (official fight time plus breaks) against an opponent of the same sex and weight division, wearing an accelerometer. Heart rate, blood lactate and ratings of perceived exertion were recorded to certify that the athletes performed the fullest.

**Results:**

The t2way test expressed differences in the athletes' BA (*p* = 0.001) and three profiles were identified: the light/middle weight male divisions, the light/middle weight female divisions and the heavy male and female ones. Athletes of all weight divisions performed their BA during the contest in all three directions (the one-sample Person's chi-square did not detect any significantly predominant one: *p* = 0.400, *p* = 0.631, *p* = 0.844, *p* = 0.749, *p* = 0.644 and *p* = 0.895, for male light, moderate and heavy, female light, moderate and heavyweight athletes, respectively). Monte Carlo method simulations suggested as the most likely scenarios those with BA involving all axes, with a slight preference of the anteroposterior and mediolateral ones.

**Discussion:**

These results suggest that the demands on judo athletes in a contest differ between weight classes and sexes.

## 1 Introduction

Judo is a Japanese martial art and an Olympic and Paralympic combat sport, introduced at the Tokyo Olympic Games in 1964 and the Seoul Olympic Games in 1988. Competitors obtain victory by throwing their opponents on their back from the standing position or by causing submission by neck choke, elbow armlock, or immobilization in ground combat (1). Contest involves open and complex skills, presents irregular intervals of effort and pause, and is characterized as an intermittent activity (2). The physiologic demands and employed metabolic mechanisms differ substantially between the dynamic and calmer phases of the contest (3), leading to specific physiologic responses with specific implications for training and conditioning and for tactical preparation (4). Several previous studies [*e.g.,* reviewed in Barreto et al. (5)] have analyzed the number of high-activity events or the duration of high-activity phases vs. calmer periods of contest. Such studies have generally been based on assessments of video materials with the subjective classifications of phases into high or low-activity classes. From these types of studies of any sport, several interesting concepts for training and competition have emerged. One of them is the “activity profile” (6–9). Overall, it refers to the individualized performance profiles of athletes through the analysis of the movement pattern that they express during competition.

Accelerometers can detect and record the body accelerations (BA) for motion along three orthogonal axes (vertical, mediolateral, and anteroposterior) in arbitrary acceleration units (a.u. or counts) (10). In this way, these devices provide a proxy measure of the mechanical work done by a moving body and wearable versions have been widely used to measure physical activity in clinical/laboratory and free-living environments (11). In combat sports, Kirk et al. (12) and Del Vecchio et al. (13) used wearable accelerometers to evaluate BA in a simulated mixed martial arts (MMA) contest and in several physical activities in a taekwondo training session (e.g., combat simulation, etc.), respectively. In addition, accelerometry has also been used in judo in the topics of injury prevention [e.g., (14–17),] and technique training [e.g. (18,19),]. However, as yet, there have been no studies quantifying in-contest BAs for judo athletes. This topic represents a potential area of exploration that would provide a valuable resource to objectively determine numerous dynamic aspects of this sport and provide a wealth of unknown information concerning the mechanical parameters of judo contestants that, alongside physiological data, might be incorporated into training programs. Of course, judo competitions are organized based on sex and weight categories, and competitors across distinct weight classes exhibit variations in technical and tactical aspects, as well as in physiology, performance, and body composition (20–22). Thus, to ensure that the fullest, most detailed picture is obtained, and for the BA data to be applied accurately, the quantification in-contest BAs for judo athletes must also be specified according to these categories.

Thus, the goals of this study were to determine the BÁs profile of the male and female weight divisions in a judo contest and to ascertain the involvement of the vertical, mediolateral, and anteroposterior axes in it, in a sample of national and international level judo athletes. It was hypothesized that two different BA profiles of the judo contest would be identified: the male and female athletes of the light and middle weight divisions and the male and female ones of the heavy weight divisions. It was also expected that athletes of all weight divisions would mainly express their BA during the contest in the anteroposterior and the vertical axes, with the BA of the anteroposterior axis being the significantly predominant one.

## 2 Material and methods

### 2.1 Design

This descriptive study was designed in accordance with the updated Declaration of Helsinki. The study protocol was approved by the Research Ethics Committee of the Principality of Asturias, Spain (No. 287/19), by the Board for Ethical Issues of the University of Innsbruck, Austria (No. 51/2019), and by the Research Ethics Committee of the University of Erlangen-Nürnberg, Germany (No. 421_19 B).

### 2.2 Participants

The studýs sample size was determined through a calculation of Coheńs statistical power analysis for the analysis of variance (ANOVA) designs (23). This process was performed using R software (www.r-project.org, version 3.3.1., 2016.06.21), setting an effect size (Coheńs f) of 0.40 and a power of 0.85. The resulting sample size was 16 participants in each group (six groups in total).

Thus, ninety-six judo athletes (average age of 22.8 ± 3.7 years, a height of 175.1 ± 10.7 cm, a body weight of 76.3 ± 17.4 kg, and a coefficient of variation [CV] of body weight of 7.8 ± 2.8%), comprising forty-eight men and forty-eight women at national and international levels, hailing from Spain, Austria, Germany, Italy, Denmark, Georgia, the Dominican Republic, Venezuela, Ukraine, and Puerto Rico, actively participated in this project. Among them were accomplished medalists from World, European, and National championships, as well as various international tournaments. The athletes of the sample were clustered on six groups, considering the official weight divisions: lightweight ([ML]; <60 kg, <66 kg, <73 kg), middleweight ([MM]; <81 kg, <90 kg) and heavyweight ([MH]; <100 kg, >100 kg) for males, and lightweight ([FL]; <48 kg, <52 kg, <57 kg), middleweight ([FM]; <63 kg, <70 kg) and heavyweight ([FH]; <78 kg, >78 kg) for females. The athletes’ body weight and height assessment was carried out with a medical scale equipped with moving weights and a stadiometer.

The recruitment phase for the study extended from September 2019 to December 2019, while data collection occurred between January 2020 and September 2022. All participants provided informed written consent. Inclusion criteria stipulated that athletes had to be active adults (≥18 years) competing at the national or international level for a minimum of four consecutive years, and free from any musculoskeletal injury or disease that could impede study completion. Overall, the competitors' weekly training programs included five to six sessions. Three to four sessions focused on technical-tactical training, while two to three emphasized conditioning, encompassing both gym-based and mat-based activities. Typically lasting two to two and a half hours, training occurred regularly for eleven months each year. At the time of the project, participants were in the preparatory phase of their training programs, with no individuals undergoing rapid weight loss. They were instructed not to use alcohol or any other drug for at least 24 h before the evaluations and to maintain their habitual diet. Characteristics of the participants are shown in Table 1.

**Table 1:** Characteristics of the participants.

Outcomes	Male (*n *= 48)			Female (*n *= 48)		
	Light weight (*n *= 16)	Middle weight (*n *= 16)	Heavy weight (*n *= 16)	Light weight (*n *= 16)	Middle weight (*n *= 16)	Heavy weight (*n *= 16)
Age (years)	22 ± 3	26 ± 6	24 ± 4	22 ± 2	22 ± 3	23 ± 2
Height (cm)	174.5 ± 5.5	181.7 ± 5.3	188.5 ± 5.5	157.6 ± 4.5	171.5 ± 4	177.2 ± 3.5
Body weight (kg)	67.9 ± 5.9	84.9 ± 3.9	103.3 ± 10.6	51.9 ± 3.7	67.1 ± 3.3	82.4 ± 9.3
CV of the body weight (%)	8.6	4.6	10.3	7.1	4.9	11.3
Official weight division^a^	4 from <60 kg/5 from <66 kg/7 from <73 kg	9 from <81 kg/7 from <90 kg	7 from <100 kg/9 from >100 kg	6 from <48 kg/5 from <52 kg/5 from <57 kg	7 from <63 kg/9 from <70 kg	7 from <78 kg/9 from >78 kg
Nationality^a^	3 AUT/3 GER/8 ESP/2 VEN	5 AUT/6 GER/4 ESP/1 GEO	3 AUT/7 GER/5 ESP/1 GEO	6 AUT/8 ESP/ 2 ITA	3 AUT/2 GER/5 ESP/2 DEN/2 DOM/1 PUR/1 UKR	6 AUT/2 GER/4 ESP/2 ITA/2 DOM
Judo degree^a^	11 1^sr^ dan/4 2nd dan/1 3rd dan	6 1sr dan/7 2nd dan/3 3rd dan	5 1sr dan/9 2nd dan/2 3rd dan	6 1^sr^ dan/10 2nd dan	10 1sr dan/5 2nd dan/1 3rd dan	6 1sr dan/9 2nd dan/1 3rd dan
Performance level^a^	7 Nat/9 Int	9 Nat/7 Int	10 Nat/6 Int	8 Nat/8 Int	6 Nat/10 Int	11 Nat/5 Int

All values are presented as a mean and standard deviation unless otherwise stated.

CV, coefficient of variation; AUT, Austria; GER, Germany; ESP, Spain; VEN, Venezuela; GEO, Georgia; ITA, Italy; DEN, Denmark; DOM, Dominican Republic; PUR, Puerto Rico; UKR, Ukraine; Nat, national; Int, International.

^a^ Number of athletes in each weight division, nationality, judo degree and performance level, respectively.

### 2.3 Procedures

Athletes participated in one simulated contest (with standing and ground judo), which was managed according to the official competition rules, against an opponent of the same sex and official weight division wearing accelerometers, and they were instructed to perform to their fullest during it. In order to detect the primary behavioral patterns of the assessed variables in the study (the athletes' BA in the judo contest and the involvement of the vertical, mediolateral, and anteroposterior axes) an extended duration for the contest was selected. Considering that a typical judo contest is a sequence of 20–30 s effort periods interspersed with 10 s breaks (4) over 4 min (breaks excluded), resulting in a total fight time of 4.5 to 5 min, the contest time for this study was set at 5 min. To ensure a precise 5-min duration for every contest, a special rule was applied; specifically, if a participant scored an *ippon* (which concludes the contest), the contest would be restarted and allowed to continue until 5 min had elapsed. Data obtained during the contest breaks were also included in the accelerometric data for the judo contest, as they were considered to express their specific background. Nonetheless, if breaks of any contests exceeded the usual break time of the judo contests [10 s (4)], these contests were considered invalid for the analysis. National/international referees judged all contests.

The accelerometers worn by athletes were located over their belly buttons, just under the knot of their judo belt, and secured using an adjustable elastic belt. This position was chosen for two reasons. Firstly, it carried the least risk of injury for athletes. Secondly, it is close to the athlete's center of gravity. Due to the nature of judo motricity, particularly the large number of asymmetric moves involved, placing the accelerometer in a location displaced from the center of gravity would potentially produce misleading, unbalanced data. The device was mounted with its vertical axis pointing upwards, such that its inclinometer was maintained in a vertical position throughout every contest (24). The accelerometers were programmed to begin collecting data two minutes before the start of a contest with data saved in 20-min files. Additionally, heart rate (HR) was monitored during the contest and ratings of perceived exertion [RPE, Borg 6–20 scale (25)] at the end. Blood lactate (BLa) was also obtained 1 min after the end of the contest [since judo athletes express the highest BLa concentration after a contest at this time (26)], to certify that the athletes performed to their fullest and that the studýs contests would produce valid data for the scientific literature.

#### 2.3.1 Accelerometry

ActiGraph GT3X accelerometers (ActiGraphTM, Pensacola, FL, USA), which have been shown to be reliable (27), sampling at 30 Hz, were employed. They use a solid-state tri-axial accelerometer to collect motion data on three axes (vertical, mediolateral, and anteroposterior). Acceleration magnitudes were registered as a.u./counts and processed according to the Freedsońs algorithm for adults (28). Given the explosive nature of the judo contest, the scoring reports obtained were edited as follow: 3-sec epoch length for axes. The BA of the contest were collected from the accelerometers as vector magnitude (Vector Magnitude CPM, in the software of ActiGraph), the equation of which is this: VM = √(x^2 ^+ y^2 ^+ z^2^). It is presented in a.u./counts per min (cpm). BA along the three axes were also collected during the contest (axis 1-vertical- CPM, axis 2 -mediolateral- CPM, and axis 3 -anteroposterior- CPM in the software) and are also presented in a.u./cpm and as a percentage.

#### 2.3.2 HR

HR was recorded using a HR monitor (Polar S810, OY, Oulu, Finland) with a chest strap. Data were expressed in beats per min (b/min) and as a percentage of the individual maximal HR (% HRmax). HRmax was calculated using this formula: [HRmax = 208 b/min—(0.7 × age in years)] (29).

#### 2.3.3 BLa

Micro-samples of arterialized blood were obtained by puncturing the earlobe to determine BLa concentrations. They were analyzed with the Lactate Pro II analyzer (Akray-KDK, Koka, Japan). Two errors occurred during BLa data acquisition: one for an athlete in the FM division and another for an athlete in the FL division.

#### 2.3.4 RPE

The Borg 6–20 RPE scale was used to determine the athletes' perceived exertion effort (25). The scale was explained to all participants prior to the beginning of the data collection.

### 2.4 Statistical analysis

All values are shown as means and standard deviations unless otherwise indicated. The normality and homoscedasticity of the data were evaluated using the Shapiro-Wilks and Bartlett's tests, respectively (*p *> 0.05). Given that they were not verified in any dependent variable, the robust t2way test (30) was performed to assess differences among all athletes' groups, using sex (male and female) and weight (light, middle, and heavy) as factors. The robust Lincon test was used to determine the between-group differences (*p *< 0.05). When significant differences were detected in the dependent variables, effect size was calculated to determine the magnitude of differences. Rosenthal's r was used (where *r* = Z/√N and “Z” value was obtained from the Exact Wilcoxon-Mann-Whitney test) and defined as small (*r* > 0.20), moderate (*r* > 0.50), or great (*r* > 0.80) (31). One-sample Pearson's chi-squared (*χ*^2^) test was used to evaluate the over- or under- representation of the BA of the three axes in the judo contest, with the a.u./cpm values expressed as percentages (to determine if any axis was significantly predominant in the contest) (*p *< 0.05). Finally, the Monte Carlo method, which simulates real sceneries and their probabilities (32), was carried out since the Pearson's chi-squared (*χ*^2^) test did not detect any significant predominant axis in the contest. Thereto, 30.000 contest simulations were carried out (10.000 for each of the vertical, mediolateral, and anteroposterior axes) in each weight division, to estimate the behavior of the motion data of the contest on three axes. It was done using a Poisson distribution with the means -as percentages- of the motion data of the contest on three axes obtained in this study. Three scenarios (and their probabilities of happening) were calculated for each axis in all divisions aiming to simulate situations corresponding to the median and the upper and lower values of the 95% confidence interval of each data distribution. All data were analyzed using R software.

## 3 Results

**Figure 1:**
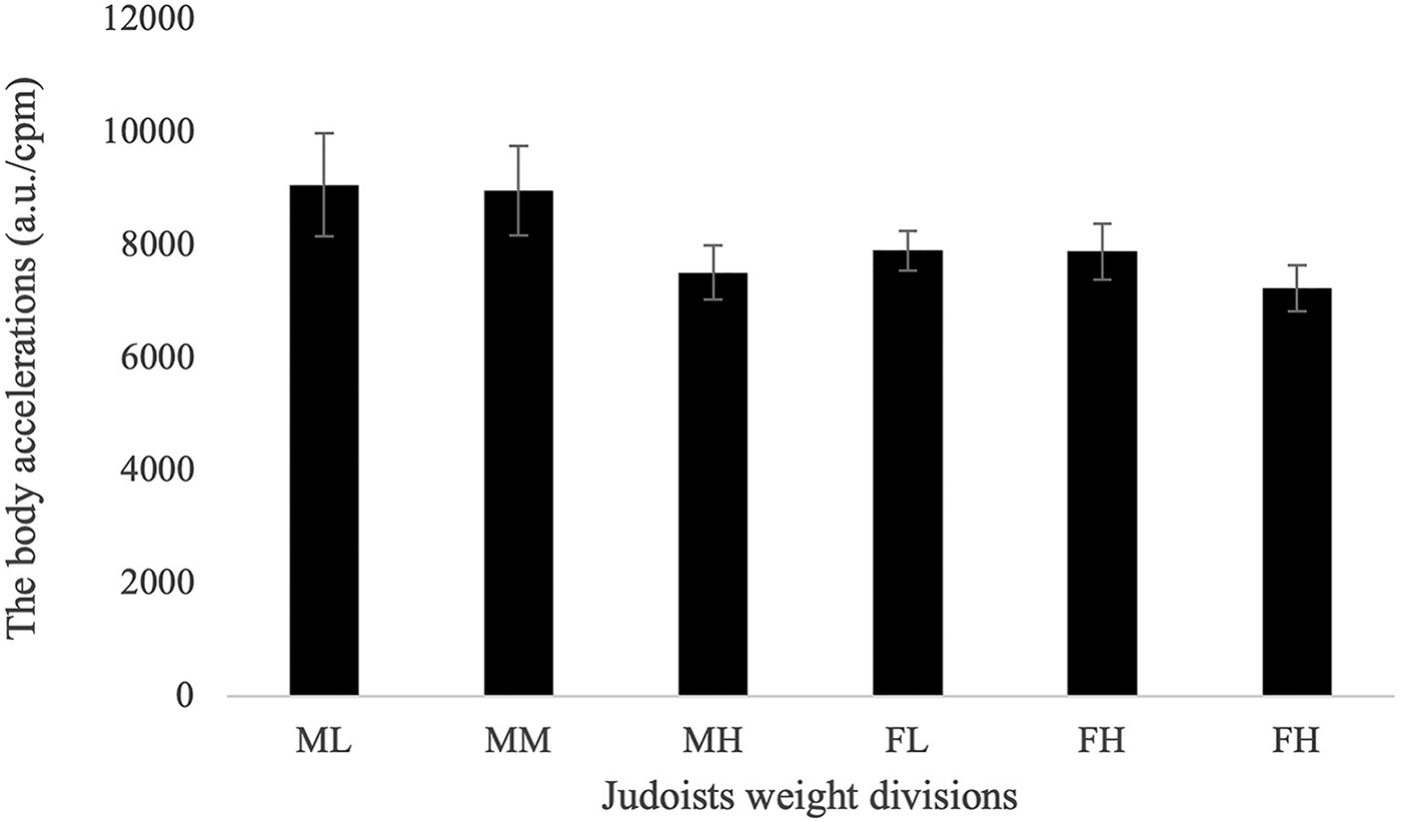
The body accelerations expressed by the whole judo weight divisions in the contest. All values are presented as a mean and standard deviation unless otherwise stated. a.u./cpm, arbitrary units-counts per min; ML, male lightweight; MM, male middleweight; MH, male heavyweight; FL, female lightweight; FM, female middleweight; FH, female heavyweight.

With respect to the BA, a significant sex-by-weight interaction was found (Table 2), with the highest BA score found for the ML category. The highest BA value for women was recorded for the FL category, this being slightly higher than the lowest male value. Large and statistically significant differences were found between BAs for ML and MH (*p *< 0.001, *r* = 0.73, moderate effect), MM and MH (*p *< 0.001, *r* = 0.73, moderate effect), FL and FH (*p *< 0.001, *r* = 0.68, moderate effect), and FM and FH (*p *= 0.003, *r* = 0.61, moderate effect) (Table 2, Figure 1).

**Table 2:** Accelerometric and physiological data of the judo contest.

Outcomes	Male			Female			Stats (*p*-values)			Effect Size (r)
	Light Weight (ML)	Middle Weight (MM)	Heavy Weight (MH)	Light Weight (FL)	Middle Weight (FM)	Heavy Weight (FH)	Sex Effect	Weight Effect	Sex by weight Effect	
BA (a.u./cpm)^a^	9,075.5 ± 915.4	8,970.3 ± 791.3	7,519.6 ± 478.6	7,908.6 ± 354.7	7,894.7 ± 497.7	7,240.3 ± 411.7	**0**.**001**	**0**.**001**	**0**.**004**	ML vs. MH, *r *= 0.73 ML vs. FL, *r *= 0.62 ML vs. FM, *r *= 0.60 ML vs. FH, *r *= 0.80 MM vs. MH, *r *= 0.73 MM vs. FL, *r *= 0.66 MM vs. FM, *r *= 0.59 MM vs. FH, *r *= 0.81 MH vs. FL, *r *= 0.42 FL vs. FH, *r *= 0.68 FM vs. FH, *r *= 0.61
BA Vertical Axis [a.u./cpm^a^ (%)]	4,588.2 ± 1,028.1 (29.3 ± 4.3)	4,672.1 ± 539.7 (30.2 ± 1.7)	3,985.4 ± 387.2 (30.8 ± 2.4)	4,180.9 ± 316.9 (30.5 ± 2.3)	4,249.2 ± 529.7 (31.4 ± 2.5)	3,982.1 ± 523.9 (31.5 ± 2.5)	**0**.**039**	**0**.**002**	0.407	MM vs. MH, *r *= 0.66 MM vs. FL, *r* = 0.44 MM vs. FH, *r* = 0.59
BA Mediolateral Axis [a.u./cpm^a^ (%)]	4,786.5 ± 522.3 (31.1 ± 3.5)	4,946.7 ± 459.5 (32.1 ± 1)	4,361.3 ± 367.8 (33.7 ± 2.1)	4,455.2 ± 209.3 (32.6 ± 1.4)	4,583.9 ± 358.5 (33.7 ± 1.7)	4,116.7 ± 305.2 (33.2 ± 1.6)	**0**.**001**	**0**.**001**	0.219	ML vs. MH, *r* = 0.44 ML vs. FH, *r* = 0.57 MM vs. MH, *r* = 0.58 MM vs. FL, *r* = 0.58 MM vs. FH, *r* = 0.73 FL vs. FH, *r* = 0.62 FM vs. FH, *r* = 0.67
BA Anteroposterior Axis [a.u./cpm^a^ (%)]	6,137.9 ± 591.2 (39.6 ± 2.2)	5,828.9 ± 573.2 (37.7 ± 2.1)	4,606.4 ± 364.8 (35.5 ± 1.2)	5,061.6 ± 367.2 (36.9 ± 1.8)	4,725.3 ± 247.8 (34.9 ± 1.7)	4,402.1 ± 327.3 (35.3 ± 3.1)	**0**.**001**	**0**.**001**	**0**.**001**	ML vs. MH, *r* = 0.83 ML vs. FL, *r* = 0.73 ML vs. FM, *r* = 0.83 ML vs. FH, *r* = 0.84 MM vs. MH, *r* = 0.80 MM vs. FL, *r* = 0.63 MM vs. FM, *r* = 0.82 MM vs. FH, *r* = 0.83 MH vs. FL, *r* = 0.55 FL vs. FM, *r* = 0.43 FL vs. FH, *r* = 0.73 FM vs. FH, *r* = 0.53
Maximum HR measured in the contest (b/min)^a^	170 ± 9	173 ± 14	177 ± 14	177 ± 13	180 ± 12	182 ± 5	**0**.**021**	0.097	0.303	—
Maximum HR measured in the contest as % HRmax (%)^a^	88 ± 5	91 ± 7	92 ± 7	92 ± 6	93 ± 7	95 ± 3	0.059	**0**.**038**	0.331	ML vs. FH, *r* = 0.59
BLa 1 min after the end of the contest (mmol/L)^a^	11.8 ± 3.1	14.3 ± 4.1	12.4 ± 2.5	8.9 ± 1.8	10.6 ± 3.1	10.2 ± 1.6	**0**.**001**	**0**.**011**	0.250	MM vs. FM, *r* = 0.46 MM vs. FH, *r* = 0.55 MH vs. FL, *r* = 0.73 MH vs. FH, *r* = 0.56
RPE at the end of the contest (a.u.)^a^	15.9 ± 2.1	16.7 ± 1.8	17.1 ± 1.4	16.6 ± 1.9	16.7 ± 2.5	17.8 ± 1.5	0.269	0.529	0.971	—

All values are presented as a mean and standard deviation unless otherwise stated.

BA, body accelerations; a.u./cpm, arbitrary units-counts per min; HR, heart rate; % HRmax, percentage of individual maximal HR; BLa, blood lactate; RPE, ratings of perceived exertion.

BLa values represents 94 data points (two errors occurred; one in the female lightweight group and another in the female middleweight).

^a^ Robust statistical/method assessments used. Roshentaĺs r effect size.

Bold values express significant differences (*p*<0.05).

**Figure 2:**
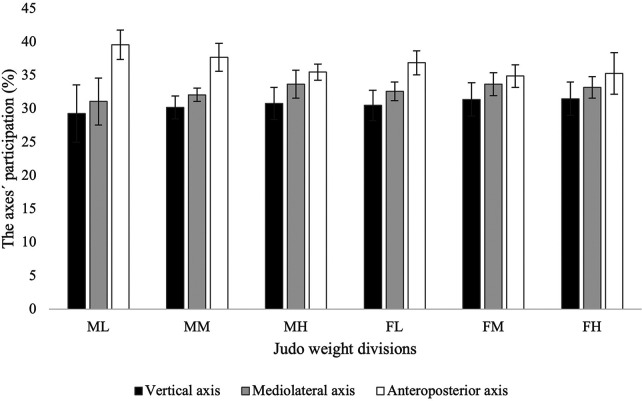
The percentage participation of the axes in the judo contest of the whole judo weight divisions. All values are presented as a mean and standard deviation unless otherwise stated. ML, male lightweight; MM, male middleweight; MH, male heavyweight; FL, female lightweight; FM, female middleweight; FH, female heavyweight.

Regarding the involvement of the three axes, a significant sex-by-weight interaction was observed for the anteroposterior axis (Table 2). Athletes of all weight divisions mainly performed their BA in the anteroposterior and the mediolateral axes, with athletes of both sexes in the light and middle weight divisions having higher BA values than those in the heavy division (Table 2, Figure 2). Differences among weight divisions in the three axes were also observed. However, the one-sample Pearson's chi-squared (*χ*^2^) test did not show any of the axes to be significantly predominant in any weight division during the contest (*p *= 0.400, *p *= 0.631, *p *= 0.844, *p *= 0.749, *p *= 0.644, and *p *= 0.895, for ML, MM, MH, FL, FM, and FH, respectively). The Monte Carlo method suggested the scenarios with BA involving all axes as the most likely, with a slight preference of the anteroposterior and mediolateral ones, across all weight divisions for both sexes (Table 3). Thus, scenarios in which BA shows a clearly dominant axis appear to have only a marginal probability of occurrence (Table 3).

**Table 3:** Results of the monte carlo simulations for the axes’ participation in the judo contest.

Axes	Possible scenario	ML (Male lightweight)	MM (Male middleweight)	MH (Male heavyweight)	FL (Female lightweight)	FM (Female middleweight)	FH (Female heavyweight)
Vertical	Upper (probability)	40% (2.3%)	41% (2.4%)	42% (2.2%)	42% (1.9%)	43% (1.9%)	43% (2%)
	Median (probability)	29% (47.4%)	30% (46.5%)	30% (51.5%)	30% (48.9%)	31% (47.8%)	31% (49%)
	Lower (probability)	19% (2.8%)	20% (3.2%)	20% (2.5%)	20% (2.9%)	21% (3.3%)	21% (3.1%)
Mediolateral	Upper (probability)	41% (2.3%)	44% (1.8%)	46% (1.7%)	44% (2.3%)	45% (2.5%)	45% (1.9%)
	Median (probability)	31% (45.2%)	32% (45.9%)	34% (43.4%)	32% (49.5%)	33% (50.1%)	33% (45.7%)
	Lower (probability)	21% (3.7%)	21% (2.5%)	23% (3.4%)	22% (3.3%)	23% (3.3%)	22% (2.8%)
Anteroposterior	Upper (probability)	52% (2.3%)	51% (1.6%)	48% (1.8%)	44% (2%)	47% (2%)	47% (2.4%)
	Median (probability)	39% (49.2%)	37% (51%)	35% (48.6%)	36% (49.5%)	35% (44.4%)	35% (47.6%)
	Lower (probability)	28% (3.4%)	26% (2.7%)	24% (2.7%)	25% (2.8%)	24% (3.4%)	24% (2.9%)

Each table cell shows the values of the axis participation expressed as percentage (and its probability of happening as percentage in brackets).

The upper and lower probability scenarios correspond to the upper and lower bounds of the 95% confidence interval of the observed data distribution.

Concerning physiological variables, the HR at the end of the contest did not manifest a significant difference among the weight divisions, the % HRmax at the end of the contest presented one significant difference between the ML and FH (*p *< 0.001, *r* = 0.59, moderate effect), the BLa 1 min after the end of the contest also showed several significant differences (MM *vs.* FM, *p *= 0.043, *r* = 0.46, moderate effect; MM *vs.* FH, *p *= 0.009, *r* = 0.55, moderate effect; MH *vs.* FL, *p *< 0.001, *r* = 0.73, moderate effect; MH *vs.* FH, *p *< 0.008, *r* = 0.56, moderate effect), and the RPE at the end of the contest did not display significant differences (Table 2).

## 4 Discussion

The goals of this study were to determine the BA profile of the judo contest of the male and female weight divisions and to ascertain the involvement of the vertical, mediolateral and anteroposterior axes in it. Results allow the identification of three BA profiles in judo; one for ML and MM, another one for FL and FM and the third one for MH and FH. In relation to the axes' involvement, findings revealed that athletes of all weight divisions tend not to favor any particular axis during the contest, since all of them are almost equally involved. The Monte Carlo method simulations suggested as the most likely scenarios those with BA involving all axes, with a slight preference of the anteroposterior and mediolateral ones.

The hypothesis that two different BA profiles of the judo contest would be identified was not confirmed; three different BA profiles were detected. To date, no study has determined the BA of the judo male and female contests. Kirk et al. (12) determined the external load experienced by the MMA athletes during a simulated MMA contest comprising three rounds of 5 min with 1 min rest between rounds, from the magnitudes of accelerations in the mediolateral, vertical and anteroposterior axes (224.32 ± 26.59 a.u./counts). External load or load are the concepts used in Sports Sciences to refer to the BA (reported as a.u./counts) of a specific physical activity. In the study of Kirk et al. (8), athletes wore Minimax X3, triaxial accelerometers secured to the torso using in a neoprene harness over the T3–4 vertebrae (the epoch configuration was not specified). Del Vecchio et al. (13) determined the BA of a sample of non-adult taekwondo athletes (expressed as VM) in several physical activities, in a taekwondo training session (e.g., combat simulation, etc.). They used an ActiGraph wGT3X+ device that was located on at the waist of the athletes, with a 5-epoch configuration and with the Freedson's algorithm for children (33) to process data and their result in the contest simulation was a BA of 530.44 ± 240.42 a.u./cpm. Given the underlying mechanisms driving the differences of BA between light, middleweight, and heavy divisions and between sexes found in the present study, they remain unknown. Thus, further research is needed to ascertain the factors contributing to these phenomena. However, these results suggest firstly, that weight affects the dynamics of the contest and secondly, that the way it influences the dynamics of the contest varies between sexes. A study by Sterkowicz-Przybycień et al. (21) with elite judo athletes, found significant differences in combat-phase times dependent on weight category and sex divisions. Additionally, in a study of male and female judo cadets, Miarka et al. (34) identified sex differences in the frequencies and timings of judo combat actions, types of techniques used, and numbers of penalties received. Thus, it seems likely that the strategic priorities in judo differ across weight divisions and between the sexes and this goes some way to explaining the results of the present study.

About the contribution of three axes in the judo contests, the hypothesis that athletes of all weight divisions would mainly express their BA during the contest in the anteroposterior and the vertical axes, being the BA of the anteroposterior axis the significantly predominant one, was not confirmed; athletes of all sexes and weight divisions produced BA on all axes equally. This finding should be considered in the design of training programs. To the authors' knowledge, the current literature contains no studies examining the issue of BA directional demands in judo contests. Santos et al. (36) evaluated the effects of judo contest on the athletes' postural control and physiological loading before, during and after a simulated contest. They observed the greatest effects manifested on the anteroposterior axis, which is not in accordance with this study. Here again, the core processes behind these outcomes remain undiscovered; therefore, additional investigation is necessary to identity their determinants. Regarding the results of the study of Santos et al. (35) and those of the Monte Carlo method of this study, the dominant trend in the contest could be that the anteroposterior axis could have a slightly higher participation than the rest axes. Nevertheless, this tendency might vary during specific contest moments (e.g., when an athlete is guarding an advantage, emphasizing the mediolateral axis).

All physiological variables examined here are in line with results from previous studies. This suggests that the athletes who participated in this study did indeed perform to their fullest abilities during the study's contests and thus, the analysis of these contests provides a useful contribution to the scientific literature. For instance, the maximum HR measured during the contests in this work were between 170 ± 9 and 182 ± 5 b/min (88.4 ± 5.2 and 94.7 ± 2.9% of the HRmax), which compares favorably with values measured by Sbriccoli et al. (36) in senior male and female judo athletes, respectively, in simulated contests [181 ± 11 b/min (94.8% of HRmax) and 176 ± 6 (92.87% of HRmax)]. Similarly, the BLa values seen in this study (from 8.9 ± 1.8 to 14.3 ± 4.1 mmol/L) are also in agreement with previous research which, after simulated judo contests are reported as being from 7.5 ± 5.1 to 18.1 ± 4.4 mmol/L (37, 38). Lastly, end-of-contest RPE, which in this study is recorded as from 15.9 ± 2.1 to 17.8 ± 1.5 a.u. is also in the same range as the value for this variable seen in other research. For example, Chtourou et al. (39) obtained values between 14 and 14.5 a.u. for regional and national level athletes after a 5-min simulated judo contest; Stavrinou et al. (40) reported 19.1 ± 0.78 a.u. in cadets after the last of a set of four 4-min contests; and Santos et al. (35) recorded 16.1 ± 0.4 a.u. for regional and national athletes after a 7-min simulated judo contest.

### 4.1 Limitations

The present study has several limitations. Firstly, there are seven weight categories for each sex, while athletes of the present study were grouped in three for each sex, to avoid too small subgroup sizes and to achieve a higher control over the results. Thus, the accelerometric data of specific weight divisions might be not exactly the same than those of the present study. However, in the case that these differences existed, due to the sample size of this study, they would not be significant and hence, it can be considered that the present study's results express the real background of the whole specific weight divisions. Secondly, in this study, a large duration of the contest was employed since, it was aimed to detect the primary pattern of behavior of the variables under assessment. To conduct a more comprehensive analysis of the variables' behavior, future studies should consider both shorter and longer durations for the contest. And thirdly, the current study's data were collected in simulation contests. BLa values are approximately 3 mmol/L higher in real competitions than in simulations (4). Nevertheless, the current study's results of this parameter are in accordance with those of the previous ones, hence they can be considered as valid.

### 4.2 Practical applications

The results of the present study can be interpreted and applied to the training of judo athletes. Firstly, the three BA profiles presented provide a benchmark for athletes. As the BA data are presented in cpm, coaches can utilize accelerometers to measure the athlete's BA in any kind of training task (regardless its duration) and subsequently, they will be able to determine how close or far athletes were from the production of BA of a specific weight division. Secondly, coaches can also use accelerometers to identify the axes which athletes perform any kind of training task, and to design training tasks focused on; executing the special techniques (*tokui-waza*) of the athletes in a specific axis (e.g., predominantly in the mediolateral or the anteroposterior one) or on staying predominantly in a specific axis to maintain an advantage and avoid penalties (e.g., in the mediolateral axis), under the symmetrical and asymmetrical grip (*aiyotsu* and *kenka-yotsu*, respectively) conditions. Finally, not only does this study offer new opportunities to improve and personalize judo training by the incorporation of a readily-measurable mechanical parameter but it also demonstrates an application of Monte Carlo simulation methods in Sports Science.

Future studies related to the BA's profile of judo contests in the male and female weight divisions, as well as the involvement of the vertical, mediolateral, and anteroposterior axes, should assess the effect of technical/tactical and strength and conditioning training programs on the findings of the present study (specifically, the three BA profiles and equal axes involvement in the contest). These studies will enable the optimization of training protocols and enhancement of athletes' performance in competition.

## 5 Conclusions

This study suggests that demands on judo athletes in a contest, measured in terms of body accelerations, differ by sex and weight classes with three different profiles; light and middle weight males, light and middle weight females, and heavy weight for both. Hence, professionals should tailor the training regimens to accommodate these distinct profiles within the weight and gender classes. In addition, this study also suggests that judo contest motion data spans equally along the vertical, mediolateral and anteroposterior axes, with the most probably scenario of a slight preference of the anteroposterior and mediolateral axes. Thus, professionals should design training tasks which could enclose all these situations to better prepare athletes for the dynamics of contests.

## Data Availability

The raw data supporting the conclusions of this article are available on request from the corresponding author.
