# Corrigendum: In-contest body acceleration profiles for the judo male and female weight divisions

**DOI:** 10.3389/fspor.2024.1429243

**Published:** 2024-05-29

**Authors:** Luis Santos, Peter A. Federolf, Friedemann Schneider, Elena Pocecco, Javier Fernández-Río, Eliseo Iglesias-Soler, Eduardo Carballeira-Fernández, Sugoi Uriarte, Xurxo Dopico-Calvo

**Affiliations:** ^1^ University of León, Department of Physical Education and Sport, León, Spain; ^2^ Performance and Health Group, Department of Physical Education and Sport, University of A Coruna, A Coruna, Spain; ^3^ Department of Sport Science, University of Innsbruck, Innsbruck, Austria; ^4^ Department of Orthopedics and Traumatology, Medical University of Innsbruck, Innsbruck, Austria; ^5^ Department of Educational Sciences, University of Oviedo, Oviedo, Spain; ^6^ Doctorate School, Universitat Politècnica de València, Valencia, Spain

**Keywords:** human motion assessment, physical activity’s patterns, combat sports, mechanical parameters, training

A Corrigendum on In-contest body acceleration profiles for the judo male and female weight division By Santos L, Federolf PA, Schneider F, Pocecco E, Fernández-Río J, Iglesias-Soler E, Carballeira-Fernández E, Uriarte S and Dopico-Calvo X (2024). Front. Sports Act. Living 6:1372314. doi: 10.3389/fspor.2024.1372314

**Incorrect Affiliation**

In the published article, there was an error in affiliation. Instead of “^1^Department of Physical Education and Sport, University of León, León, Spain. ^2^Performance and Health Group, Department of Physical Education and Sport, University of A Coruña, A Coruña, Spain”.

It should be “^1^University of León, Department of Physical Education and Sport, León, Spain

^2^Performance and Health Group, Department of Physical Education and Sport, University of A Coruna, A Coruna, Spain”.

The authors apologize for this error and state that this does not change the scientific conclusions of the article in any way. The original article has been updated.

**Text Correction**

In the published article, there was an error in the second paragraph of 2.2. Participants and it contents. The part of the text that is in strikethrough (and in red color) must be removed from the manuscript.

Thus, ninety-six judo athletes, comprising of forty-eight men and forty-eight women at national and international levels, hailing from Spain, Austria, Germany, Italy, Denmark, Georgia, the Dominican Republic, Venezuela, Ukraine, and Puerto Rico, actively participated in this project. Among them were accomplished medalists from World, European, and National championships, as well as various international tournaments. As aforementioned, the athletes of the sample were clustered into six groups, considering the official weight divisions: lightweight [(ML)]; <60 kg, <66 kg, <73 kg],middleweight [(MM)]; <81 kg, <90 kg], and heavyweight [(MH); <100 kg, >100 kg] for males, and lightweight [(FL);<48 kg, <52 kg, <57 kg], middleweight [(FM); <63 kg, <70 kg], and heavyweight [(FH); <78 kg, >78 kg] for females. Thus, ninety-six judo athletes [average age of 22.8 ± 3.7 years, a height of 175.1 ± 10.7 cm, a body weight of 76.3 ± 17.4 kg, and a coefficient of variation (CV) of body weight of 7.8 ± 2.8%], comprising forty-eight men and forty-eight women at national and international levels, hailing from Spain, Austria, Germany, Italy, Denmark, Georgia, the Dominican Republic, Venezuela, Ukraine, and Puerto Rico, actively participated in this project. Among them were accomplished medalists from World, European, and National championships, as well as various international tournaments. The athletes of the sample were clustered on six groups, considering the official weight divisions: lightweight [(ML); <60 kg, <66 kg, <73 kg], middleweight [(MM); <81 kg, <90 kg] and heavyweight [(MH); <100 kg, >100 kg] for males, and lightweight [(FL); <48 kg, <52 kg, <57 kg], middleweight ([FM]; <63 kg, <70 kg) and heavyweight [(FH); <78 kg, >78 kg] for females. The athletes’ body weight and height assessment was carried out with a medical scale equipped with moving weights and a stadiometer.

The authors apologize for this error and state that this does not change the scientific conclusions of the article in any way. The original article has been updated.

